# Outcome of Targeted Therapy Recommendations for Metastatic and Recurrent Head and Neck Cancers

**DOI:** 10.3390/cancers12113381

**Published:** 2020-11-15

**Authors:** Hossein Taghizadeh, Robert M. Mader, Leonhard Müllauer, Thorsten Fuereder, Alexandra Kautzky-Willer, Gerald W. Prager

**Affiliations:** 1 Department of Medicine I, Clinical Division of Oncology, Medical University of Vienna, 1090 Vienna, Austria; seyed.taghizadehwaghefi@meduniwien.ac.at (H.T.); robert.mader@meduniwien.ac.at (R.M.M.); thorsten.fuereder@meduniwien.ac.at (T.F.); 2 Comprehensive Cancer Center Vienna, 1090 Vienna, Austria; leonhard.muellauer@meduniwien.ac.at; 3 Clinical Institute of Pathology, Medical University Vienna, 1090 Vienna, Austria; 4 Department of Medicine III, Division of Endocrinology and Metabolism, Medical University of Vienna, 1090 Vienna, Austria; alexandra.kautzky-willer@meduniwien.ac.at; 5 Department of Medicine III, Gender Medicine Unit, Medical University of Vienna, 1090 Vienna, Austria

**Keywords:** metastatic and recurrent head and neck cancers, targeted therapy, molecular profiling, time to treatment failure, turnaround time, immunohistochemistry, gender-specific differences, precision medicine

## Abstract

**Simple Summary:**

In this retrospective study, we aimed to provide molecular-driven therapy recommendations for patients with recurrent/metastatic head and neck cancers based on the respective individual molecular profile. For 31 of 50 patients (62.0% of all patients), a targeted therapy approach could be recommended. Therapy recommendations were significantly more often issued for men than for women. Eventually, 14 patients (28%) received the recommended targeted therapy. Six patients (12%) achieved stable disease and four patients (8%) experienced progressive disease. The median time to treatment failure was 2.8 months. Our analysis showed that although precision medicine approaches are implementable and feasible for the management of recurrent/metastatic head and neck cancers in daily clinical routine, there are major limitations and challenges that have to be overcome.

**Abstract:**

Recurrent/metastatic (R/M) head and neck cancers bear a poor prognosis. In this analysis, we examined the efficacy and the outcome of targeted therapy recommendations based on the patients’ molecular tumor portrait after failure of all standard therapy options. In this single-center, real-world retrospective analysis of our platform for precision medicine, we analyzed the molecular profile of 50 patients diagnosed with R/M head and neck cancer. Tumor samples of the patients were examined using next-generation sequencing panels of mutation hotspots, microsatellite instability (MSI) testing, and immunohistochemistry (IHC). In 31 cases (62.0% of all patients), a molecular-driven targeted therapy approach was recommended. Eventually, 14 patients (28%) received the suggested targeted therapy. Six of fourteen patients (43%) achieved stable disease conditions and four patients (29%) experienced a progressive disease. The median time to treatment failure was 2.8 months. Therapy recommendations were significantly more often issued for men (*p* = 0.037) than for women. This analysis demonstrated that precision medicine provided the basis for molecular-driven therapy recommendations in over half of the patients with advanced therapy refractory head and neck cancers, with significantly more therapy recommendations for men. Our analysis showed that although precision medicine approaches are implementable and feasible for the management of recurrent/metastatic head and neck cancers in daily clinical routine, there are major limitations and challenges that have to be overcome.

## 1. Introduction

Oral cavity, pharyngeal, laryngeal, and salivary gland cancers are classified as head and neck cancers (HNCs). Globally, the number of incidents of HNCs is more than 650,000, with over 330,000 deaths annually. Males are diagnosed significantly more often with HNC than females are, with a ratio ranging from 2:1 to 4:1 [1,2]. Several risk factors have been identified that are associated with the emergence of head and neck cancers, including excessive alcohol consumption, use of tobacco products, and human papillomavirus (HPV) infection [3,4]. Studies have shown a synergistic and multiplicative risk effect of alcohol intake and tobacco use on head and neck carcinogenesis [5].

For the treatment of head and neck cancers, a multidisciplinary and multimodal treatment concept is pursued, including surgical resection of the primary tumor, radiation, and systemic chemotherapy. In recurrent/metastatic head and neck cancer (R/M HNC), systemic palliative antineoplastic therapy is of particular importance for disease control. Despite therapeutic advances in the treatment of head and neck cancers, including the introduction of immunotherapy, mortality rates are high due to the dismal prognosis of R/M HNC at around 15 months [6]. 

In recent years, major efforts and endeavors have been undertaken to analyze the molecular tumor profile of the malignant tissue of various cancer diseases to identify actionable targets to improve the therapeutic management and thus disease control. This treatment approach is known as precision medicine. In a few particular tumor types, treatment with custom-tailored tyrosine kinase inhibitors or immunotherapeutic agents has become possible, such as trastuzumab in human epidermal growth factor receptor 2 (HER2-positive) breast cancer or gastric cancer [7,8], imatinib in KIT+ gastrointestinal stromal tumor (GIST) [9], and B-rapidly accelerated fibrosarcoma (BRAF)-directed therapy with vemurafenib or dabrafenib/trametinib in melanoma [10].

In contrast to these examples, well-studied and established biomarkers for personalized treatment approaches in head and neck cancer patients are limited at present. Targeted therapy agents have very limited activity in R/M HNC, and the current treatment strategy is still based on tumor location and disease stage rather than on tumor biology [11].

However, the investigation of the molecular profile of HNCs may help to detect and identify new targets to expand the current therapeutic armamentarium.

In this study, we conducted a retrospective subgroup analysis of all 50 patients with R/M HNC that had been enrolled and profiled in our special platform for precision medicine of the Comprehensive Cancer Centre of the Medical University of Vienna (CCC-MUV). 

We sought to map the molecular profiles of R/M HNC to identify and target specific molecular alterations. We also discuss the challenges, limitations, and the time to treatment failure (TTF) of precision medicine approaches in this patient group.

## 2. Results

From June 2013 to March 2020, a total of 50 patients diagnosed with R/M HNC were included in this subgroup analysis from the cohort of our platform for precision medicine, which, so far, has profiled over 600 patients with various advanced and therapy-refractory solid tumors. In this analysis, all patients were Caucasians, including 33 men and 17 women.

The cohort of R/M HNC comprised 18 patients with oropharyngeal and oral cancer, 16 patients with salivary gland cancer, 8 patients with nasal cavity and paranasal sinus cancer, 4 patients with nasopharyngeal cancer, and 4 patients with hypopharyngeal and laryngeal cancer.

The three most common histopathological subtypes were squamous cell carcinoma of the head and neck (HNSCC), adenocarcinoma, and adenoid-cystic carcinoma, with 24, 13, and 11 patients, respectively.

The median age at first diagnosis was 53.4 years, ranging from 23.1 to 84.2 years, and the median age at the time when the molecular profiling was performed was 59.5 years, ranging from 26.3 to 85.2 years (Table 1). The tumor tissue for molecular profiling was obtained by biopsy in 16 patients and during surgical treatment in the other 34 patients. At the time of molecular profiling, all of the 50 patients had an advanced and therapy-refractory R/M HNC with no further standard treatment options available. Forty-three of them had experienced a disease relapse, and 45 had metastases, mainly in the lungs, bones, and liver. 

The patients received a median of two lines of prior systemic chemotherapy, ranging from 1 to 6 lines. The chemotherapy regimens included TPF (docetaxel, cisplatin or carboplatin, and 5-fluorouracil), EXTREME (cetuximab, cisplatin, and 5-fluorouracil, followed by maintenance with cetuximab), PC (paclitaxel combined with carboplatin), carboplatin and gemcitabine, capecitabine, pembrolizumab combined with cisplatin and 5-fluorouracil, and nivolumab.

In total, we detected 97 mutations in 40 patients. The five most frequently mutated genes were *TP53* (*n* = 21; 21.6%), *CDKN2A* (*n* = 5; 5.1%), *PIK3CA* (*n* = 5; 5.1%), *NOTCH1* (*n* = 4; 4.1%), and *PTEN* (*n* = 4; 4.1%) that accounted for 40.2% of all mutations. No mutation was detected in 10 (20%) patients. See Table 2 and Table 3 for further details. Six gene-fusions were identified, namely, *EIF3E–RSPO2* (*n* = 2), *MYBL1–NFIB* (*n* = 2), *FNDC3B–PIK3CA*, and *MON2–RAP1GDS1*. Moreover, we detected 15 gene amplifications in six different tumor specimens, including *AR*, *CCND1* (*n* = 2), *FGF19* (*n* = 2), *FGF3* (*n* =2), *KRAS* (*n* = 2), *MYC* (*n* = 2), *CCND3*, *CCNE1, CDK6*, and *RICTOR*.

In addition to molecular alterations, immunohistochemistry (IHC) frequently detected the expression of EGFR (33 subjects; 66.0%), phosphorylated mTOR (29 subjects; 58.0%), MET (15 subjects; 30.0%), and PD-L1 (13 subjects; 26.0%). Expression rates below 20% were seen for platelet-derived growth factor receptor alpha (PDGFRA), NTRK, and C-kit receptor (KIT) in 9 (18%), 9 (18%), and 5 (10%) patients, respectively. Three male patients had an expression of the androgen receptor and two males and one female exhibited an expression of the estrogen receptor. IHC identified five patients (10%) with loss of PTEN. The loss of PTEN was subsequently verified and characterized by fluorescence in situ hybridization (FISH) as heterozygous PTEN deletions.

None of the patients had a high microsatellite instability (MSI) status. IHC or FISH could not be performed for two male patients due to insufficient tumor material.

In 34 patients, the tumor tissue was obtained during surgical resection. The median time interval between resection and molecular analysis of the tumor tissue was 13.4 months (range: 1–37 months). Sixteen patients underwent biopsy to obtain fresh tumor tissue for molecular profiling. 

In 31 cases (62.0% of all patients), a molecular-driven targeted therapy approach could be recommended. In over two-thirds of all recommendations (*n* = 22/31; 71.0%), the molecular-driven treatment approach was mainly derived from the molecular characteristics determined by immunohistochemistry. The 31 recommended targeted treatments included pembrolizumab (*n* = 7), cetuximab alone (*n* = 5), cetuximab combined with temsirolimus (*n* = 4), palbociclib (*n* = 3), androgen deprivation therapy with bicalutamide and leuprorelin (*n* = 3), and crizotinib (*n* = 2). Erlotinib, imatinib, ponatinib, poziotinib, sunitinib, cetuximab combined with paclitaxel, and temsirolimus combined with carboplatin were each proposed in one case. Table 4 describes the rationale for the recommended targeted therapy approaches. 

**Figure 1:**
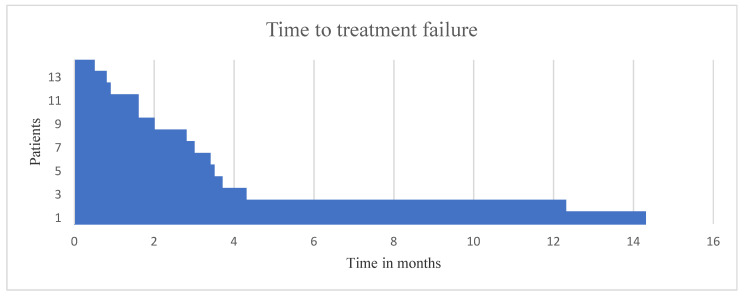
Time to treatment failure (TTF) in the 14 R/M HNC patients who received the recommended targeted therapy.

Eventually, 14 patients (28%)—comprised of twelve men (12/14; 86%) and two women (2/14; 14%)—received the recommended targeted therapy. Ten of the 14 patients underwent radiological assessment (see Table 5). Six of fourteen patients (43%) achieved stable disease and four patients (29%) experienced progressive disease. Three of six patients who achieved stable disease and three of four patients confirmed to have progressive disease were diagnosed with non-squamous cell carcinoma of the subtype salivary gland carcinoma. Four patients (29%) died before radiological assessment could be performed. The eventually applied molecular-based targeted therapies included androgen deprivation therapy (*n* = 3), cetuximab alone (*n* = 2), cetuximab combined with temsirolimus (*n* = 2), pembrolizumab (*n* = 2), crizotinib, imatinib, ponatinib, poziotinib, sunitinib, and cetuximab combined with paclitaxel. The median time to treatment failure (TTF) of the 14 patients who received the targeted therapy was 2.8 months (range: 0.4–14.2 months). Figure 1 depicts the TTF. The TTF of the patients with salivary gland carcinoma (*n*= 7) was 2.9 months. The median time interval between biopsy and completion of the molecular analysis of the tumor tissue was 24 days (range: 20–37 days). The median turnaround time between initiation of molecular profiling and discussion by the multidisciplinary team (MDT) and molecular-based therapy initiation for all 50 patients was 35 and 47 days, respectively.

Seventeen patients (34%) did not receive the offered targeted therapy. The reasons for not applying the recommended targeted agent included the following: rapid deterioration of performance status (*n* = 14), death of patient (*n* = 1), and the treating oncologist favored another treatment regimen due to the overall clinical situation of the patients or refusal of any further treatment, including targeted therapy options (*n* = 2).

Nineteen patients (38%) did not harbor an actionable molecular target. Two patients were lost to follow-up. Seven patients died prior to therapy initiation. Eventually, ten patients (20%) received an experimental therapy, based on clinical data provided by phase 1 and phase 2 trials. The TTF in these ten patients was 2.5 months (range: 0.5–13.8 months). The experimental therapies included axitinib (*n*= 2), lenvatinib (*n*= 2), pembrolizumab (*n*= 2), nivolumab, bortezomib, vinorelbine, and conventional chemotherapy with cisplatin^+^, cyclophosphamid^+^, doxorubicin (CAP). The median turnaround time between the failure of the last standard therapy regimen and the initiation of the experimental therapy was 13 days.

**Figure 2:**
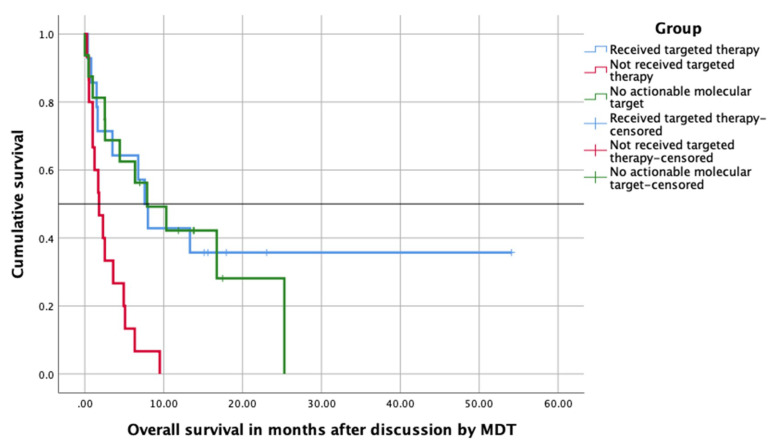
Kaplan–Meier curves showing overall survival after discussion by the multidisciplinary team (MDT) in three different R/M patient groups.

**Figure 3:**
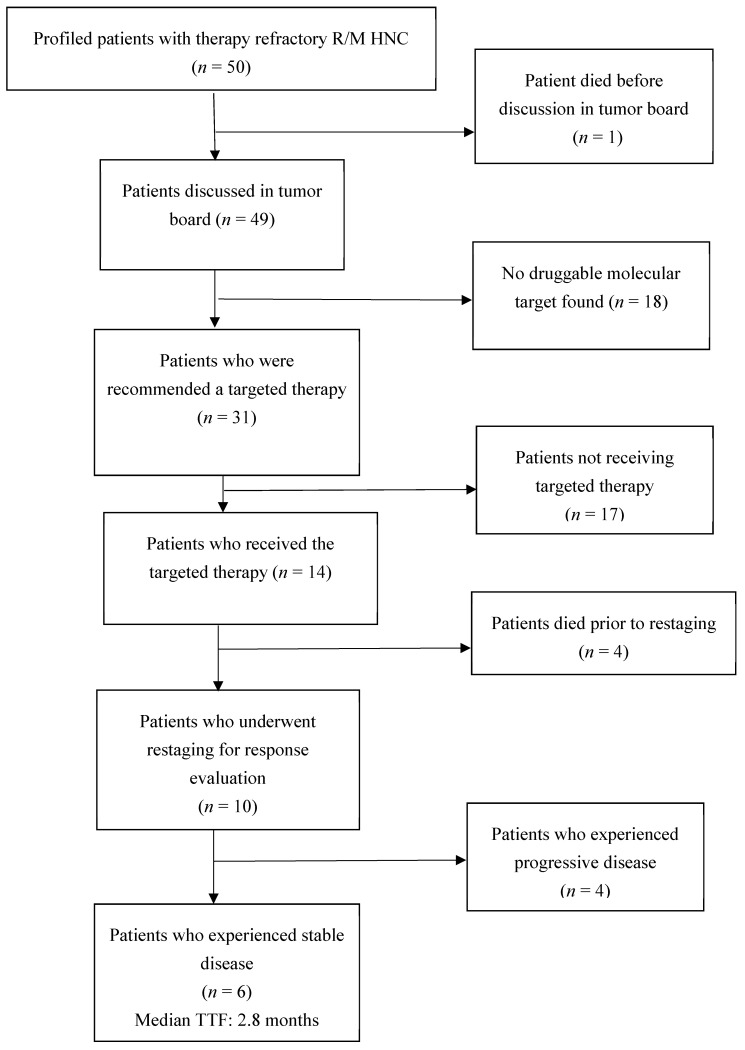
Flow chart of the 50 R/M HNC patients.

We compared the overall survival (OS) of the aforementioned three groups, i.e., patients receiving the targeted therapy (group 1), patients not receiving the targeted therapy (group 2) and patients without any actionable molecular targets (group 3) and depicted the survival data by using Kaplan–Meier survival curve. The shortest median overall survival (mOS) was seen in patients who did not receive the targeted therapy. The mOS of patients who received experimental therapy was slightly longer than patients receiving the targeted therapy (7.9 months versus 7.6 months). See Figure 2 for overall survival and Figure 3 for patients’ flow.

Further, the Fisher’s exact test revealed that targeted therapy recommendations were significantly more often (*p* = 0.035) issued for men (24/31) than for women (7/31). In our cohort, loss of PTEN (*n* = 5) only occurred in male patients. PDGFRA expression was seen in eight male patients, but only in one female patient. According to the Fisher’s exact test, the gender-specific differences regarding loss of PTEN (*p* = 0.146) and PDGFRA expression (*p* = 0.131) were not statistically significant.

In 34 patients, the tumor tissue was obtained during surgical resection. The median time interval between resection and molecular analysis of the tumor tissue was 13.4 months (range: 1–37 months). Sixteen patients underwent biopsy to obtain fresh tumor tissue for molecular profiling. The median time interval between biopsy and completion of the molecular analysis of the tumor tissue was 24 days (range: 20–37 days). The median turnaround time between initiation of molecular profiling and discussion by the MDT and molecular-based therapy initiation for all 50 patients was 35 and 47 days, respectively.

## 3. Discussion

In this study, we showed, for the first time, the clinical applicability, feasibility, limitations, and gender-specific differences of molecular-based treatment approaches in 50 R/M HNC patients with no further available standard treatment option in the real world.

Currently, standard systemic therapy for patients with R/M HNC includes systemic cytotoxic chemotherapy and monoclonal antibodies, including the EGFR inhibitor cetuximab and the PD-1 checkpoint inhibitors such as pembrolizumab and nivolumab. Regarding chemotherapy, the backbone of most chemotherapy regimens is formed by the platinum agents cisplatin and carboplatin, whereby carboplatin is less neurotoxic, nephrotoxic and ototoxic than cisplatin. In addition, 5-fluorouracil and the taxanes docetaxel, paclitaxel are important chemotherapeutics agents [12]. Further, immunotherapy is becoming increasingly important. The seminal clinical phase 3 trial KEYNOTE-048 demonstrated the superiority of pembrolizumab combined with a platinum and 5-flouoruacil in terms of overall survival when compared with cetuximab in conjunction with a platinum and 5-fluorouracil 13.0 months versus 10.7 months). For those R/M HNSCC patients with high PD-L1 expression pembrolizumab alone significantly prolonged OS compared with cetuximab plus a platinum and fluorouracil combination [6]. Similarly, the phase 3 trial Checkmate-141 tested nivolumab against single-agent investigator’s choice of therapy (methotrexate, docetaxel, or cetuximab). The researchers reported that nivolumab significantly improved OS compared to chemotherapy (7.7 months versus 5.1 months) [13].

In our MTD, for 31 patients, a targeted molecular-driven therapy option was recommended. The majority of the targeted treatment suggestions were mainly derived from the molecular characteristics determined by immunohistochemistry. Thus, our analysis demonstrated and underscored the major clinical relevance of immunohistochemistry in devising targeted therapy recommendations.

Moreover, we observed significantly more targeted therapy recommendations for male patients than for female patients. Although the gender-specific differences regarding the PDGFRA expression and the loss of PTEN were not statistically significant, these results suggest gender-specific differences in the molecular profile. Thus, these findings warrant further intense research.

There are several limitations to this study, as well as in the implementation and integration of molecular-based treatment strategies in daily clinical practice. For 31 patients (62%), a targeted therapy was offered. Eventually, fourteen patients (28%) received the targeted therapy. Although our study demonstrated that precision medicine can be implemented and integrated into clinical practice, only six of fourteen patients (12%) achieved stable disease and clinically benefitted from the personalized treatment approach. Four patients experienced progressive disease and another four patients died prior to radiological assessment. The TTF was relatively short with only 2.8 months, only slightly longer than patients who were given experimental therapy with 2.5 months TTF. The mOS of patients treated with targeted therapy was even slightly shorter than those patients with experimental therapy (7.6 months versus 7.9 months).

One important reason for the poor outcome may be the long median turnaround time of 47 days between molecular profiling and therapy initiation. Without an effective and potent antitumoral treatment, the cancer disease can further spread and advance, leading to health deterioration. Thus, during this time interval, 15 patients died or their overall health condition rapidly worsened before therapy initiation, despite being in a stable health condition with an Eastern Cooperative Oncology Group performance status (ECOG PS) of 0/1 before this time interval. In contrast, the turnaround time in patients treated with experimental therapy was only 13 days.

One way to reduce the long turnaround time may be the use of liquid biopsy for R/M HNC patients to generate the molecular information in a shorter period of time [14,15]. Further, the development of faster gene sequencing machines and the employment of automated immunohistochemistry may contribute to a faster molecular analysis [16,17]. Another way to shorten this time interval is to obtain the molecular information of HNC before the failure of the last line of standard treatment.

Another important limitation is the relatively long median turnaround time of 13.4 months between resection and molecular profiling of the tumor specimen obtained during resection in over half of the patients (*n* = 34, 68%). Due to the dynamic nature of cancer diseases, the tumor biology of the tumor may have changed in this time interval, which would mean that the molecular profile might differ at the time point of the initiation of the molecular analysis from the time point of surgical resection. Consequently, the matching of the targeted therapy to the molecular profile might be not accurate. In future, liquid biopsy may be a suitable tool to obtain a current molecular profile of the tumor to devise an accurate targeted therapy concept.

Another reason for the relatively modest outcome may be the complex intra- and inter-tumoral molecular heterogeneity of HNC. The included HNC subtypes other than HNSCC, including adenocarcinoma, adenoid cystic carcinoma, and mucoepidermoid carcinoma added to the heterogeneity since these subtypes differ from HNSCC in terms of molecular patterns [18,19]. Further, the number of patients was limited (*n* = 50) for this heterogeneous group of HNC patients. A further limitation is that this study was retrospective.

Currently, the long turnaround time between molecular profiling and therapy initiation limits the efficacy of precision medicine approaches in R/M HNC.

The observed mutations and expressions in R/M HNC patients were in keeping with previous studies [20,21]. The ten most frequent mutations (*TP53, CDKN2A, PIK3CA, NOTCH1, PTEN, ATM, BRCA2, CREBBP, EGFR,* and *MET*) together accounted for more than 50% percent of all detected mutations. The rest of the detected mutations were of low frequency (<5%) and demonstrated the well-known molecular heterogeneity and diversity of HNC [22,23,24].

One strategy to minimize the chance of resistance to cancer therapy is the application of safe combination therapies with regard to the molecular tumor profile [25]. Another important reason may be that—because of the long turnaround time—there was not enough time for the targeted therapy to reveal its full potential.

Currently, the multicenter clinical phase II trial UPSTREAM of the European Organisation for Research and Treatment of Cancer (EORTC) (EudraCT Number 2017-000086-74, NCT03088059) is the first biomarker-based umbrella trial in R/M HNC. Treatment stratification and decisions are based on next-generation sequencing, and immunohistochemistry umbrella trials test different targeted therapy agents in a single cancer type [11].

## 4. Methods

### 4.1. Patients and Design of the Precision Medicine Platform

Patients with pretreated R/M HNC who had progressed to all standard therapies confirmed by the Response Evaluation Criteria in Solid Tumors 1.1 (RECIST 1.1) were eligible for enrollment in our precision medicine platform, provided tissue samples were available for molecular profiling. The tumor samples were either obtained by a fresh tumor biopsy performed by physicians from the Department of Interventional Radiology at the Medical University of Vienna (Vienna, Austria) or were provided by the archives of the Department of Pathology at the Medical University of Vienna when tumor biopsy was not possible. Only patients were included who had an Eastern Cooperative Oncology Group (ECOG) status of 0 or 1. Our platform for precision medicine is not a clinical trial, but it seeks to provide targeted therapy recommendations for patients for whom no standard therapy option is available. All patients in this analysis had to be at least 18 years of age at the time of molecular profiling and had to give informed consent prior to enrollment in our platform. This analysis was approved by the Institutional Ethics Committee of the Medical University of Vienna (No. 1039/2017). The General Hospital of Vienna directly covered all costs for the molecular analysis and targeted therapy, provided the cancer patients had no further standard treatment options.

### 4.2. Tissue Samples

Formalin-fixed, paraffin-embedded tissue samples from patients with R/M HNC who had progressed to all standard therapy regimens were obtained from the archive of the Department of Pathology, Medical University of Vienna, Austria.

### 4.3. Cancer Gene Panel Sequencing

DNA was extracted from paraffin-embedded tissue blocks with a QIAamp Tissue KitTM (Qiagen, Hilden, Germany). Then, 10 ng DNA per tissue sample was provided for sequencing. The DNA library was created by multiplex polymerase chain reaction with the Ion AmpliSeq Cancer Hotspot Panel v2 (Thermo Fisher Scientific, Waltham, MA, USA), which covers the mutation hotspots of 50 genes. The panel includes driver mutations, oncogenes, and tumor suppressor genes. By mid-2018, the gene panel was expanded using the 161-gene next-generation sequencing panel of Oncomine Comprehensive Assay v3 (Thermo Fisher Scientific, Waltham, MA, USA), which covers genetic alterations and gene fusions. The Ampliseq cancer hotspot panel was sequenced with an Ion PGM (Thermo Fisher Scientific, Waltham, MA, USA) and the Oncomine Comprehensive Assay v3 on an Ion S5 sequencer (Thermo Fisher Scientific, Waltham, MA, USA). The generated sequencing data were analyzed afterward with the help of the Ion Reporter Software (Thermo Scientific Fisher Scientific, Waltham, MA, USA). We referred to the BRCA Exchange, ClinVar, COSMIC, dbSNP, OMIM, and 1000 genomes for variant calling and classification.

### 4.4. Immunohistochemistry

Immunohistochemistry (IHC) was performed using 2 μm-thick tissue sections read by a Ventana Benchmark Ultra stainer (Ventana Medical Systems, Tucson, Arizona, USA). The following antibodies were applied: anaplastic lymphoma kinase (ALK) (clone 1A4; Zytomed, Berlin, Germany), CD20 (clone L26; Dako Omnis from Agilent Technologies, Santa Clara, CA, USA), CD30 (clone BerH2; Agilent Technologies, Vienna, Austria), DNA mismatch repair (MMR) proteins, including MLH1 (clone M1, Ventana Medical Systems, Oreo Valley, AZ, USA), PMS2 (clone EPR3947, Cell Marque, Rocklin, CA, USA), MSH2 (clone G219-1129, Cell Marque, Rocklin, CA, USA), and MSH6 (clone 44, Cell Marque, Rocklin, CA, USA), epidermal growth factor receptor (EGFR) (clone 3C6; Ventana Medical Systems, Tucson, Arizona, USA), estrogen receptor (clone SP1; Ventana Medical Systems, Tucson, Arizona, USA), human epidermal growth factor receptor 2 (HER2) (clone 4B5; Ventana Medical Systems, Tucson, Arizona, USA), HER3 (clone SP71; Abcam, Cambridge, UK), C-kit receptor (KIT) (clone 9.7; Ventana Medical Systems, Tucson, Arizona, USA), MET (clone SP44; Ventana Medical Systems, Tucson, Arizona, USA), NTRK (clone EPR17341, Abcam, Cambridge, UK), phosphorylated mammalian target of rapamycin (p-mTOR) (clone 49F9; Cell Signaling Technology, Danvers, Massachusetts, USA), platelet-derived growth factor alpha (PDGFRA) (rabbit polyclonal; Thermo Fisher Scientific, Waltham, MA, USA), platelet-derived growth factor alpha beta (PDGFRB) (clone 28E1, Cell Signaling Technology, Danvers, Massachusetts), programmed death-ligand 1 (PD-L1) (clone E1L3N; Cell Signaling Technology until mid-2018, but as of mid-2018, the clone BSR90 from Nordic Biosite, Stockholm, Sweden was used), progesterone receptor (clone 1E2; Ventana Medical Systems, Tucson, Arizona, USA), phosphatase and tensin homolog (PTEN) (clone Y184; Abcam, Cambridge, UK), and ROS1 (clone D4D6; Cell Signaling Technology, Danvers, Massachusetts, USA).

To assess the immunostaining intensity for the antigens EGFR, p-mTOR, PDGFRA, PDGFRB, and PTEN, a combinative semiquantitative score for immunohistochemistry was used. The immunostaining intensity was graded from 0 to 3 (0 = negative, 1 = weak, 2 = moderate, and 3 = strong). To calculate the score, the intensity grade was multiplied by the percentage of the corresponding positive cells: (maximum 300) = (% negative × 0) + (% weak × 1) + (% moderate × 2) + (% strong × 3).

The immunohistochemical staining intensity for HER2 was scored from 0 to 3+ (0 = negative, 1+ = negative, 2+ = positive, and 3+ = positive), pursuant to the scoring guidelines of the Dako HercepTest from the company Agilent Technologies (Agilent Technologies, Santa Clara, CA, USA). In the case of HER2 2+, a further test with HER2 in situ hybridization was performed to verify the HER2 gene amplification.

Estrogen receptor and progesterone receptor stainings were graded according to the Allred scoring system from 0 to 8. MET staining was scored from 0 to 3 (0 = negative, 1 = weak, 2 = moderate, and 3 = strong) based on a paper by Koeppen et al. [26].

For PD-L1 protein expression, the tumor proportion score was calculated, which is the percentage of viable malignant cells showing membrane staining. In addition, since 2019, the expression is also determined by the combined positive score.

ALK, CD30, CD20, and ROS1 staining were classified as positive or negative based on the percentage of reactive tumor cells—however, without graduation of the staining intensity. In ALK- or ROS1-positive cases, the presence of a possible gene translocation was evaluated by fluorescence in situ hybridization (FISH).

The status of microsatellite instability (MSI) was analyzed by the MSI Analysis System, Version 1.1 (Promega Corporation, Madison, WI, USA).

### 4.5. Fluorescence In Situ Hybridization (FISH)

FISH was applied only in selected cases to verify PTEN loss. FISH was performed with 4 μm-thick formalin-fixed, paraffin-embedded tissue sections. The following FISH probes were utilized: PTEN (10q23.31)/Centromere 10 (ZytoVision, Bremerhaven, Germany). Two hundred cell nuclei per tumor were evaluated. The PTEN FISH was considered positive for PTEN gene loss of ≥30% of cells with only one or no PTEN signals. A chromosome 10 centromere FISH probe served as a control for ploidy of chromosome 10.

### 4.6. Multidisciplinary Team for Precision Medicine

After careful and extensive examination of the molecular profile of each tumor sample by an experienced molecular pathologist, the results were discussed in a multidisciplinary team (MDT) meeting, which was held every two weeks.

Regular attendees of the MDT included molecular pathologists, radiologists, clinical oncologists, surgical oncologists and basic scientists. The MDT recommended targeted therapy based on the individual molecular portrait of each patient. Targeted therapies included tyrosine kinase inhibitors, checkpoint inhibitors (e.g., anti-PD-L1 monoclonal antibodies) and growth factor receptor antibodies with or without endocrine therapy. MDT treatment recommendations were prioritized from high to low depending on the level of evidence, following Phase III to Phase I studies.

In cases where more than one actionable molecular alteration was detected, MDT suggested a therapy strategy that targets as many molecular aberrations as possible, with special regard to the toxicity profile of the individual anti-tumor agents and their potential interactions. Since all patients received all available state-of-the-art therapies for their solid tumors before being included in our platform for precision medicine, almost all targeted therapies were proposed as off-label use. When a patient’s tumor profile and clinical characteristics met the requirements for inclusion in a recruiting clinical trial for targeted therapies at our cancer center, patients were asked whether they wanted to participate in the respective study conducted in compliance with ethical and regulatory guidelines.

### 4.7. Study Design and Statistics

The Fisher’s exact test was employed to explore potential gender-specific differences regarding the therapy recommendation rate and the molecular profile. A *p*-value less than 0.05 was considered as statistically significant. For the examination of overall survival, we used Kaplan–Meier survival curves. For statistical analysis, the software package IBM SPSS Statistics Version 26 was used.

## 5. Conclusions

Taken together, the management of R/M HNC poses several major challenges, including the long turnaround time until therapy initiation and the intra- and inter-tumoral molecular heterogeneity of HNC.

Our analysis showed that precision medicine approaches can be of clinical benefit in selected heavily pretreated R/M HNC. However, the overall benefit was limited and the TTF was relatively modest. Thus, further intense research is warranted to further develop precision medicine in the management of R/M HNC patients.

## Figures and Tables

**Table 1:** Patient characteristics (*n* = 50).

Patient Characteristics	Number
Median (range) age at first diagnosis	53.4 (23.1–84.2)
Median (range) age at molecular profiling	59.5 (26.3–85.2)
Male patients	33 (66%)
Female patients	17 (34%)
Caucasian	50 (100%)
Hypopharyngeal and laryngeal cancer	4 (8%)
Nasopharyngeal cancer	4 (8%)
Nasal cavity and paranasal sinus cancer	8 (16%)
Oropharyngeal and oral cancer	18 (36%)
Salivary gland cancer	16 (32%)
Relapsed disease	43 (86%)
Metastatic disease	45 (90%)
Systemic chemotherapy received	50 (100%)
Prior chemotherapy regimens	1–6
Therapy recommendations for patients For male patients For female patients	31 (62%) 24 (48%) 7 (14%)
Histopathological subtypes Squamous cell carcinoma Adenocarcinoma Adenoid cystic carcinoma Mucoepidermoid carcinoma	24 (48%) 14 (28%) 11 (22%) 1 (2%)

**Table 2:** Genomic profile of the recurrent/metastatic head and neck cancer (R/M HNC) patients.

Mutated Genes	Number of Mutations	Percentage of Occurrence in Patients (*n* = 50)	Percentage of all Mutations (97 Mutations in Total)
*TP53*	21	42.0%	21.6%
*CDKN2A*	5	10.0%	5.2%
*PIK3CA*	5	10.0%	5.2%
*NOTCH1*	4	8.0%	4.1%
*PTEN*	4	8.0%	4.1%
*ATM*	3	6.0%	3.1%
*BRCA2*	3	6.0%	3.1%
*CREBBP*	3	6.0%	3.1%
*EGFR*	3	6.0%	3.1%
*MET*	3	6.0%	3.1%
*APC*	2	4.0%	2.1%
*RAD51D*	2	4.0%	2.1%
*RET*	2	4.0%	2.1%
*SMAD4*	2	4.0%	2.1%
*SMARCA4*	2	4.0%	2.1%
*TET2*	2	4.0%	2.1%
*AKT2*	1	2.0%	1.0%
*CDK2*	1	2.0%	1.0%
*CDK12*	1	2.0%	1.0%
*CDKN2B*	1	2.0%	1.0%
*CTNNB1*	1	2.0%	1.0%
*ERBB2*	1	2.0%	1.0%
*FANCD2*	1	2.0%	1.0%
*FBXW7*	1	2.0%	1.0%
*FGFR1*	1	2.0%	1.0%
*FGFR2*	1	2.0%	1.0%
*HRAS*	1	2.0%	1.0%
*KRAS*	1	2.0%	1.0%
*MAX*	1	2.0%	1.0%
*MDM2*	1	2.0%	1.0%
*MSH6*	1	2.0%	1.0%
*MYCN*	1	2.0%	1.0%
*NF1*	1	2.0%	1.0%
*NFE2L2*	1	2.0%	1.0%
*NOTCH2*	1	2.0%	1.0%
*NOTCH3*	1	2.0%	1.0%
*NRAS*	1	2.0%	1.0%
*PALB2*	1	2.0%	1.0%
*RAD50*	1	2.0%	1.0%
*RAD51B*	1	2.0%	1.0%
*RHOA*	1	2.0%	1.0%
*RNF43*	1	2.0%	1.0%
*SF3B1*	1	2.0%	1.0%
*SLX4*	1	2.0%	1.0%
*STK11*	1	2.0%	1.0%
*TSC1*	1	2.0%	1.0%
*TSC2*	1	2.0%	1.0%

**Table 3:** Detailed information of mutations of each R/M HNC patient.

Patient	Mutations
Squamous cell carcinoma	
1	RB1: c.949C > G, missense mutation
2	CDKN2A: c.172C > T: nonsense mutation (stop-gain); TP53: c.481G > A: missense mutation
3	PIK3CA: c.3140A > G: missense mutation; TP53: c.848C > G: missense mutation
4	EGFR; FBXW7; NOTCH1; SMAD4; TP53 *
5	0
6	ATM: c.5185G > C: missense mutation; TP53: c.583A > T: missense mutation; TP53: c.467G > C: missense mutation
7	TP53: c.820G > T: missense mutation
8	CDKN2A: c.164delG: deletion; TP53: c.488A > G: missense mutation
9	TP53: c.833C > T: missense mutation
10	0
11	TP53: c.880G > T: nonsense mutation; TP53: c.1006G > T: missense mutation; NOTCH1: c.1127G > A: missense mutation; ATM: c.2899C > A: missense mutation
12	VHL: c.362G > A: missense mutation; PTEN: c.301C > T: nonsense mutation (stop-gain)
13	PIK3CA: c.162G > A: missense mutation; TP53: c.472delC: deletion
14	CDKN2B: c.256G > A: missense mutation; BRCA2: c.2374T > C: missense mutation; RAD51B: c.520A > G: missense mutation; RNF43: c.319G > A: missense mutation; NOTCH3: c.3257A > C: missense mutation
15	0
16	PIK3CA: c.1633G > A: missense mutation; MET: c.1076A > G: missense mutation; TP53: c.722C > T: missense mutation
17	TET2: c.5103G > A: missense mutation; TSC1: c.3181A > C: missense mutation; TET2: c.3703G > A: missense mutation; BRCA2: c.3355G > C: missense mutation
18	MSH6: c.4001 + 10_4001 + 13delTAAC: deletion; CREBBP: c.1537C > A: missense mutation; ERBB2: c.2033G > A: missense mutation
19	CDKN2A: c.341C > T: missense mutation
20	EGFR *
21	TP53: c.472C > T: missense mutation; TP53: c.455C > T: missense mutation; MET: c.504G > T: missense mutation; FANCD2: c.4270A > G: missense mutation; NF1: c.3547C > G: missense mutation; SMARCA4: c.4105C > T: missense mutation
22	TP53: c.1024C > T: nonsense mutation; CDKN2A: c.83_100delTGCGGGCGCTGCTGGAGG: deletion; MYCN: c.849G > T: missense mutation; SLX4: c.2087A > G: missense mutation
23	RHOA: c.14G > A: missense mutation; PIK3CA: c.3140A > G: missense mutation; CDK2: c.391C > T: missense mutation; NFE2L2: c.80A > T: missense mutation
24	HRAS: c.38G > A: missense mutation; SMAD4: c.1558G > T: nonsense mutation (stop-gain)
Non-squamous cell carcinoma	
1	0
2	0
3	0
4	TP53: c.742C > T: missense mutation
5	APC: c.3920T > A: missense mutation
6	BRCA2: c.6770C > G: missense mutation
7	FGFR2: c.755C > G: missense mutation; NOTCH1: c.7397delC: deletion; EGFR: c.2884C > T: missense mutation; CREBBP: c.4303G > A: missense mutation; RAD51D: c.26G > C: missense mutation
8	RET: c.2372A > T: missense mutation
9	PIK3CA: c.3140A > G: missense mutation; RAD51D: c.992T > A: missense mutation; CDK12: c.3052G > A: missense mutation
10	CTNNB1: c.110C > G: missense mutation; TP53: c.818G > A: missense mutation; NOTCH2: c.2543G > T: missense mutation; RET: c.2372A > T: missense mutation
11	RAD50: c.980G > A: missense mutation; PALB2: c.1001A > G: missense mutation
12	0
13	PTEN: c.633C > G: missense mutation
14	KRAS: c.34G > A: missense mutation; STK11: c.587G > T: missense mutation; TSC2: c.65G > A: missense mutation
15	TP53: c.637C > T: nonsense mutation
16	ATM: c.9142C > G: missense mutation
17	MAX: c.66delT: deletion; CREBBP: c.785G > T: missense mutation
18	0
19	0
20	APC: c.4298C > T: missense mutation
21	TP53: c.376T > G: missense mutation; MET: c.3029C > T: missense mutation
22	NRAS; TP53 *
23	PTEN; TP53 *
24	SF3B1: c.1874G > A: missense mutation; PTEN: c.1078A > G: missense mutation; SMARCA4: c.3484G > A: missense mutation
25	NOTCH1: c.5912C > A: missense mutation; MDM2: c.1242A > C: missense mutation; AKT2: c.1544C > T: missense mutation
26	CDKN2A: c.151_155delGTCAT: deletion; FGFR1: c.478_480delGAT: deletion

* No detailed information available due to insufficient documentation.

**Table 4:** Rationale for targeted therapy recommendations (*n* = 31).

Therapeutic Agent (trading name)	Targets	Overview of Current FDA Approval in Different Entities	Overview of Current EMA Approval in Different Entities	Number of Recommended and Received Cases, Responses
Pembrolizumab (Keytruda)	PD-1 and hypermutability	Melanoma, NSCLC, HNSCC, HL, urothelial carcinoma, microsatellite instability-high cancer, gastric cancer, and cervical cancer	Melanoma, NSCLC, HNSCC, HL, and urothelial carcinoma	Recommended for 7 patients with PD-L1 expression Applied in 2 patients: 1 patient died before restaging and 1 patient achieved SD for 3 months
Cetuximab (Erbitux)	EGFR	CRC and HNSCC	CRC and HNSCC	-Recommended for 5 patients with EGFR expression and *KRAS* wildtype Applied in 2 patients: 1 patient achieved SD for 12 months and 1 patient died before restaging -Recommended in combination with temsirolimus for 4 patients with EGFR expression and *KRAS* wildtype, as well as loss of PTEN and mTOR expression Applied in 2 patients: 1 patient died before restaging and 1 patient experienced PD -Recommended in combination with paclitaxel for 1 patient with EGFR expression and *KRAS* wildtype Patient achieved SD for 3 months
Palbociclib (Ibrance)	CDK4 and CDK6	HER2-negative breast cancer	HER2-negative breast cancer	Recommended for 3 patients with *CDKN2A* mutation
Bicalutamide (Casodex)	AR	Prostate cancer	Prostate cancer	Recommended in combination with leuprorelin for 3 patients with AR expression Applied in 2 patients who experienced PD
Leuprorelin (Trenantone)	GNRHR	Prostate cancer	Prostate cancer	See bicalutamide
Crizotinib (Xalkori)	ALK, ROS1, and MET	ROS1^+^ or ALK^+^ NSCLC	ROS1^+^ or ALK^+^ NSCLC	Recommended for 2 patients with MET expression Applied in 1 patient who died before restaging
Erlotinib (Tarceva)	EGFR	NSCLC and PDAC	NSCLC and PDAC	Recommended for 1 patient with EGFR mutation
Imatinib (Gleevec)	ABL1, BCR, KIT, and PDGFR	Ph^+^ CML, KIT^+^ GIST, MDS/MPD associated with PDGFR, and Ph^+^ALL	Ph^+^ CML, KIT^+^ GIST, MDS/MPD associated with PDGFR, and Ph^+^ALL	Recommended for and applied in 1 patient with KIT expression, who achieved SD for 13 months
Ponatinib (Iclusig)	ABL1, BCR, FGFR, FLT3, KIT, and PDGFR	CML and Ph^+^ALL	CML and Ph^+^ ALL	Recommended for and applied in 1 patient with *FGFR3* and *FGFR19* gene amplification, who achieved SD for 4 months
Poziotinib (No trading name yet)	EGFR and HER2	Experimental application but no approval	Experimental application but no approval	Recommended for 1 patient with exon 20 insertion mutation Applied in 1 patient, who achieved SD for 3 months
Sunitinib (Sutent)	PDGFR, KIT, VEGFR, RET, and FLT3	RCC, PDAC, and GIST	RCC, PDAC, and GIST	Recommended for and applied in 1 patient with KIT expression, who experienced PD
Temsirolimus (Torisel)	mTOR	RCC	RCC and MCL	Recommended in combination with carboplatin for 1 patient with mTOR expression and loss of PTEN Please see also cetuximab

ABL1, Abelson murine leukemia viral oncogene homolog 1; AML, acute myeloid leukemia; ALK, anaplastic lymphoma kinase; ALL, acute lymphatic leukemia; AR, androgen receptor; BCR, breakpoint cluster region; CML, chronic myeloid leukemia; CRC, colorectal cancer; EGFR, epidermal growth factor receptor; EMA, European Medicines Agency; FDA, Food and Drug Administration; FGFR, fibroblast growth factor receptor; FLT3, FMS-like tyrosine kinase 3; GIST, gastrointestinal stromal tumor; GNRHR, gonadotropin-releasing hormone receptor; HER2, human epidermal growth factor receptor 2; HL, Hodgkin lymphoma; HNSCC, head and neck squamous cell carcinoma; KIT, C-kit receptor; MCL, mantle cell lymphoma; MDS, myelodysplastic syndrome; MPD, myeloproliferative disorder; mTOR, mammalian target of rapamycin; NSCLC, non-small-cell lung carcinoma; PD, progressive disease; PD-1, programmed cell death protein 1; PDAC, pancreatic ductal adenocarcinoma; PDGFR, platelet-derived growth factor receptor; PD-L1, programmed death-ligand 1; Ph+, Philadelphia chromosome-positive; p-mTOR, phosphorylated mammalian target of rapamycin; PTEN, phosphatase and tensin homolog; RCC, renal cell carcinoma; RET, rearranged during transfection; SD, stable disease; VEGFR, vascular endothelial growth factor.

**Table 5:** Characteristics of the 14 R/M HNC patients receiving the molecular-based targeted therapy recommendation.

Number Gender Entity Histology	Detected Mutations	Immunohistochemistry	Applied Targeted Therapy	Age at Molecular Profiling	TTF in Months	Therapy Response	Cause of Therapy Termination
1 Male Salivary gland cancer Adenoid cystic carcinoma	*APC*	EGFR score = 115, PTEN score = 100, p-mTOR score = 35, KIT score = 125, MET score = 1, PDGFRA score = 80, and NTRK score = 100	Imatinib	46.43	14.2	SD	PD
2 Female Salivary gland cancer Adenoid cystic carcinoma	*MAX* and *CREBBP*	EGFR score = 300, PTEN score = 160, p-mTOR score = 30, PDGFRA score = 80, and NTRK score = 210	Cetuximab alone	80.02	12.2	SD	PD
3 Male Salivary gland cancer Adenocarcinoma	*CTNNB1,* *NOTCH2,* *RET,* *SLX4,* and *TP53*	EGFR score = 210, PTEN score = 90, and NTRK score = 100	Ponatinib	54.95	4.2	SD	PD
4 Male Salivary gland cancer Adenocarcinoma	*CDK12,* *PIK3CA,* and *RAD51D*	EGFR score = 280, PTEN score = 190, and p-mTOR score = 100	Cetuximab + paclitaxel	47.94	3.6	SD	n.a. *
5 Male Paranasal sinus cancer Squamous cell carcinoma	*EGFR* and *TP53*	Not performed	Poziotinib	62.86	3.4	SD	n.a. *
6 Male Hypopharyngeal cancer Squamous cell carcinoma	*TP53*	EGFR score = 300, PTEN score = 80, and p-mTOR = 260	Pembrolizumab	59.04	3.3	SD	ECOG PS > 2
7 Male Salivary gland cancer Adenocarcinoma	*CREBBP,* *EGFR,* *FGFR2,* *NOTCH1,* and *RAD51D*	PDGFRA score = 20, EGFR score = 300, NTRK score = 110, PTEN score = 300, and AR score = 250	Androgen deprivation therapy with bicalutamide and leuprorelin	36.72	2.9	PD	PD
8 Male Oropharyngeal cancer Squamous cell carcinoma	*PTEN*	EGFR score = 210, MET score = 1, p-mTOR = 150, and loss of PTEN	Cetuximab + temsirolimus	59.41	2.7	PD	PD
9 Male Salivary gland cancer Adenocarcinoma	0	EGFR score = 250, PTEN score = 90, p-mTOR = 50, and AR score = 200	Androgen deprivation therapy with bicalutamide and leuprorelin	47.87	1.9	PD	PD
10 Male Salivary gland cancer Adenocarcinoma	*TP53*	EGFR score = 30, MET score = 1, PDGFRA score = 100, and p-mTOR = 70	Sunitinib	65.51	1.5	PD	PD
11 Male Oropharyngeal cancer Adenocarcinoma	*TP53*	EGFR score = 300, PDGFRA score = 20, PTEN score = 130, p-mTOR score = 95, and NTRK score = 45	Cetuximab alone	73.61	1.5	n.a.	Death
12 Female Hypopharyngeal cancer Squamous cell carcinoma	*FANCD2,* *MET,* *NF1,* *NOTCH1,* and *SMAD4*	PTEN score = 90 and PD-L1-positive (TPS = 5 and CPS = 10)	Pembrolizumab	36.61	0.8	n.a.	Death
13 Male Salivary gland cancer Adenocarcinoma	0	EGFR score = 120, MET score = 1, p-mTOR = 230, and AR score = 200	Androgen deprivation therapy with bicalutamide and leuprorelin	82.55	0.7	n.a.	Death
14 Male Oropharyngeal cancer Squamous cell carcinoma	*CDKN2A* and *TP53*	EGFR score = 240, MET score = 1, p-mTOR = 50, and loss of PTEN	Cetuximab + temsirolimus	64.27	0.4	n.a.	Death

* Therapy still ongoing; n.a., not applicable; AR, androgen receptor; CPS, combined prognostic score; ECOG PS, Eastern Cooperative Oncology Group performance status; EGFR, epidermal growth factor receptor; PD, progressive disease; PD-L1, programmed death-ligand 1; PDGFRA, platelet-derived growth factor receptor alpha; p-mTOR, phosphorylated mammalian target of rapamycin; SD, stable disease, PTEN, phosphatase and tensin homolog; TPS, tumor-positive score; TTF, time to treatment failure.
